# The SAMe-TT_2_R_2_ Score Predicts Warfarin Control in an Australian Population with Atrial Fibrillation

**DOI:** 10.3390/jcm8060882

**Published:** 2019-06-20

**Authors:** Nijole Bernaitis, Gemma Clark, Sarah Kohja, Stephanie Leong, Shailendra Anoopkumar-Dukie

**Affiliations:** 1 School of Pharmacy & Pharmacology and Quality Use of Medicines Network, Griffith University, Queensland 4222, Australia; gemma.clark@griffithuni.edu.au (G.C.); s.dukie@griffith.edu.au (S.A.-D.); kohjas@myumanitoba.ca (S.K.); leongs@myumanitoba.ca (S.L.); 2 School of Pharmacy, University of Manitoba, Winnipeg, MB R3T 2N2, Canada

**Keywords:** oral anticoagulant, warfarin, risk model, SAMe-TT_2_R_2_ Score

## Abstract

Background: Warfarin requires regular monitoring with the time in therapeutic range (TTR), a common indicator of control and TTR > 70% is indicative of efficient anticoagulation. The SAMe-TT_2_R_2_ (sex, age, medical history, treatment, tobacco use, race) model has been utilised as a predictor of warfarin control, with a score ≥ 2 indicative of poor control. However, it has been suggested that race may be over-represented in this model. To date, no Australian studies have applied this model, possibly because race is not routinely recorded. Therefore, the aim of this study was to apply the SAMe-TT_2_R_2_ model in an Australian population on warfarin managed by both a warfarin care program (WCP) and general practitioner (GP). Methods: Retrospective data was collected for patients receiving warfarin via a WCP in Queensland and whilst being managed by a GP. Patient data was used to calculate the SAMe-TT_2_R_2_ score and the TTR for each patient. Mean TTR was used for analysis and comparison with the categorised SAMe-TT_2_R_2_ score. Results: Of the 3911 patients managed by a WCP, there was a significantly lower mean TTR for patients with a SAMe-TT_2_R_2_ score ≥ 2 compared to 0–1 (78.6 ± 10.7% vs. 80.9 ± 9.5%, *p* < 0.0001). Of these patients, 200 were analysed whilst managed by a GP and the categorised SAMe-TT_2_R_2_ score did not result in a statistically different mean TTR (69.3 ± 16.3% with 0–1 vs. 63.6 ± 15.0% with ≥2, *p* = 0.089), but a score ≥2 differentiated patients with a TTR less than 65%. Conclusions: The SAMe-TT_2_R_2_ model differentiated Australian patients with reduced warfarin control, despite the exclusion of race. In Australia, the SAMe-TT_2_R_2_ score could assist clinicians in identifying Australian patients who may obtain reduced warfarin control and benefit from additional interventions such as a dedicated WCP.

## 1. Introduction

Warfarin is a vitamin K antagonist that has long been used as an oral anticoagulant for the prophylaxis and treatment of stroke in patients with atrial fibrillation (AF) [1]. However, warfarin has a narrow therapeutic index and requires continuous monitoring of the international normalised ratio (INR) with a target INR range between 2.0 to 3.0 being generally recommended [2]. Warfarin control is commonly measured by the time in therapeutic range (TTR) with values above 70% recommended to minimise adverse events [2]. Since the development of non-vitamin K oral anticoagulants (NOACs) that do not require monitoring, health practitioners are faced with the challenge of determining the most effective anticoagulant for the patient [3]. Australian AF guidelines [4] suggest patients with a warfarin TTR < 60% should be considered for NOAC therapy. However, it has also been reported that almost 5% of Australian patients that switch from warfarin to NOACs subsequently revert to warfarin therapy [5]. Therefore, assistance with the decision of the most suitable oral anticoagulant agent and whether a patient can achieve and maintain good warfarin control is critical [6].

Predictor stroke and bleed risk models are commonly used to assist health practitioners in prescribing anticoagulation in patients with AF [7]. Studies have applied these stroke and bleed risk models for the dual purpose of predicting warfarin control [8,9], but several predictor models specifically identifying warfarin control have been developed [10,11,12,13]. In 2013, Apostalakis et al. [13] proposed the SAMe-TT_2_R_2_ (sex female, age < 60 years, medical history (more than two of: hypertension, diabetes, coronary artery disease/myocardial infarction, peripheral artery disease, congestive heart failure, previous stroke, pulmonary disease, hepatic or renal disease), treatment with interacting medication of amiodarone, tobacco use (doubled), race (doubled)) model as a predictor of warfarin control, with a score ≥ 2 indicative of poor control. Several guidelines for AF [14,15,16] have incorporated the SAMe-TT_2_R_2_ model as the recommended tool to predict potential warfarin control and aid decisions regarding warfarin therapy. Current Australian AF guidelines [4] do not include recommendations regarding this model, and to date, no studies have been found in Australian populations, possibly because race is not routinely recorded in Australian medical records. However, Fauchier et al. [17] suggested some factors, particularly non-White race and tobacco use, may be over-represented depending on the setting. In support of this, Lee et al. [18] modified the model by removing race and tobacco and demonstrated a good prediction of TTR with this modified score. Similarly, Gallego et al. [19] found a reduced discriminatory value of the SAMe-TT_2_R_2_ score in an anticoagulation clinic without any prevalence of differing ethnicity, while Skov et al. [20] demonstrated the score was not predictive of TTR in a specialised anticoagulant clinic achieving high-quality control, i.e., mean TTR 76%. Therefore, given the conflicting data regarding the application of the SAMe-TT_2_R_2_ model in the absence of race or predominantly Caucasian populations, the aim of this study was to investigate the predictive ability of the SAMe-TT_2_R_2_ model in an Australian population receiving warfarin management from different management models, namely a specialist warfarin care program (WCP) and a general practitioner (GP).

## 2. Materials and Methods

### 2.1. Data Collection

Ethics approval PHM/09/14/HREC was obtained from Griffith University. Retrospective data was collected for patients enrolled in a WCP at Sullivan Nicolaides Pathology, Queensland, as of September 2014. The WCP manages each patient’s warfarin therapy after referral from their GP. The service includes INR testing, interpretation of results, and provision of information relating to dosing and the time of next appointment.

Patients taking warfarin therapy for AF were identified and data was collected, including sex, age, medical history, and concurrent medication. Tobacco use was noted for a few patients but not actively recorded by the program, and race was not available for any patient. Dates and results of INR tests were collected for the entire time patients were managed by the WCP. Patient records were further screened to determine whether INR results were available for the time prior to enrolment in the WCP, i.e., whilst managed by their GP. Patients were excluded from further analysis if there were no INR results prior to enrolment in the WCP or continuous results were unavailable. Additional exclusions for the time managed by the GP included INR target ranges not being documented or mixed INR ranges documented.

### 2.2. Statistical Analyses

Patient-specific TTR was calculated using the Rosendaal et al. [21] linear interpolation method with software from INR Pro^®^. TTR was calculated for all patients whilst managed by the WCP and for the time managed by the GP where available. Exclusions were made if insufficient INR results, i.e., less than 30 days of testing, were available. The SAMe-TT_2_R_2_ score [13] was calculated for all eligible patients and the sum of the score was used to categorise patients. Patient characteristics were represented as numbers and percentages and categorical data were represented as mean ± standard deviation. Comparison of risk scores were made using non-parametric methods via the Mann–Whitney and Kruskal–Wallis test. Statistical analysis was performed using GraphPad Instat software version 3 (GraphPad Software, Inc., La Jolla, CA, USA) with *p* < 0.05 considered significant.

## 3. Results

Of the 3954 patients being treated for AF at the WCP, a total of 3911 patients were included in the study following exclusions for having less than 30 days of INR tests (43 patients). There were 2010 (51.4%) male and 1901 (48.6%) female patients (Table 1). The majority (96.2%) of patients were over 60 years of age with the mean age being 78.2 ± 9.4 years. The mean TTR was 81.0 ± 9.6% whilst managed by the WCP. There was a significant difference (*p* < 0.001) between the mean TTR of patients with a SAMe-TT_2_R_2_ score of 0–1 (80.9 ± 9.5%) compared to patients with a SAMe-TT_2_R_2_ score ≥ 2 (78.6 ± 10.7%) (Table 2).

Of the 3911 patients included in the study, there were 200 patients with INR results available whilst being managed by their GP. This was following exclusion for no INR results being available prior to management by the WCP (3557), continuous results unavailable (83), less than 30 days of INR tests (52), and INR target range either not recorded or recorded as mixed (19). The mean TTR was 68.5 ± 16.2% whilst managed by the GP, which was significantly lower (*p* < 0.001) than the TTR whilst managed by the WCP. Once patients managed by the GP were categorised according to SAMe-TT_2_R_2_ score, the mean TTR of patients with a score of 0–1 (69.3 ± 16.3%) compared to patients with a score ≥ 2 (63.6 ± 15.0%) was not significantly different (*p* = 0.089) (Table 2).

## 4. Discussion

The SAMe-TT_2_ R_2_ model was proposed to assist clinicians in identifying patients that may not achieve good warfarin control, as identified by a score ≥ 2 [13]. Meta-analyses [22] have shown the score to be predictive of TTR, but to our knowledge there have been no studies in an Australian population where race is not routinely recorded. Therefore, the aim of this study was to apply the SAMe-TT_2_R_2_ model in an Australian population. This study found a SAMe-TT_2_R_2_ score ≥ 2 was associated with significantly lower TTR in patients managed by a WCP. However, in a subset of these patients, when managed by their GP, no significant difference in mean TTR was found according to the SAMe-TT_2_R_2_ category but a score ≥ 2 was associated with a mean TTR < 65%.

In this study, the mean TTR was 81% whilst managed by the WCP and 69% whilst managed by the GP. Previous work by this research team compared two models of Australian warfarin management and produced similar results plus further investigated possible factors contributing to the differences in control [23]. Similar to this, recent Australian observational studies have reported warfarin TTR from 69% to 81% [9,24,25] depending on the management model. High levels of warfarin control were also found in the large comparative studies of warfarin and the NOACs in Australian populations of 73% to 76% [26,27,28]. Comparable to this, the mean TTR for patients whilst managed by the WCP was 81%. In these patients, a SAMe-TT_2_R_2_ score ≥ 2 was associated with a significantly lower mean TTR; however, this mean TTR remained above 78%. Similarly, Gallego et al. [19] associated a high SAMe-TT_2_R_2_ score with poorer TTR in an anticoagulation clinic but the TTR remained above 70% with a score ≥ 3. In contrast to these studies, Skov et al. [20] investigated the SAMe-TT_2_R_2_ model in an anticoagulant clinic with a mean TTR of 76% and found it was not predictive of TTR. Both the study by Gallego et al. [19] and our study was in an anticoagulation clinic without differing ethnicity as a factor. Previous studies [17,18] have suggested that race may be over-represented in the SAMe-TT_2_R_2_ model. Further to this, Caucasian race has commonly been correlated with higher TTR [10,29,30,31,32], including in two Australian studies that demonstrated poorer warfarin control in Indigenous Australians [33,34]. Despite this, race is not routinely collected by medical services in Australia. It may be assumed that the majority of patients in Australia are Caucasian but statistics show around one third of Australians are born overseas [35]. Therefore, whilst the SAMe-TT_2_R_2_ model was predictive of reduced control in patients with a score ≥ 2, this study was unable to analyse the impact on ethnicity on the discriminatory value of the SAMe-TT_2_R_2_ score. Future Australian studies could identify race and apply this as a factor to the SAMe-TT_2_R_2_ model and further determine the impact of ethnicity in the Australian context. Similarly, tobacco use was not widely reported in this population. Both Fauchier et al. [17] and Lee et al. [18] also cautioned against the possible over-representation of tobacco use in the SAMe-TT_2_R_2_ model, so the influence of this factor on outcomes requires further exploration in future studies.

In this study the SAMe-TT_2_R_2_ score did not provide a significant difference in the mean TTR in the subset of patients with data available whilst being managed by their GP. However, a SAMe-TT_2_R_2_ score ≥ 2 was associated with a TTR less than 65%, whilst patients with a score of 0–1 had a mean TTR above 65%. Current Australian AF guidelines [4] suggest consideration should be given to changing to a NOAC in patients with a warfarin TTR < 60%. However, the Australasian Society of Thrombosis and Haemostasis [36] define patients stably anticoagulated on warfarin as a TTR > 65% over a three-month period. Therefore, whilst the SAMe-TT_2_R_2_ score did not find a statistical difference in mean warfarin TTR for GP-managed patients in this study, the categorisation according to the SAMe-TT_2_R_2_ score may still be clinically significant by identifying patients who may be suitable for warfarin therapy according to a recommended minimum TTR of 65%. In 2018, Zulkifly et al. [37] reviewed nineteen studies applying the SAMe-TT_2_R_2_ score and concluded that it adequately predicted good or poor warfarin control, but the definition of good control varied between 60% and 70%. Our study found the SAMe-TT_2_R_2_ score predictive of 65% warfarin TTR. In contrast Poli et al. [38] found a SAMe-TT_2_R_2_ score > 2 to be a good predictor of poor control but described this as a TTR < 70%. Similarly, Pastori et al. [39] associated an increased SAMe-TT_2_R_2_ score with a reduced number of patients with TTR < 70%. Therefore, the predictive ability of the SAMe-TT_2_R_2_ score may be dependent on the determined acceptable level of warfarin control in particular settings. Several authors [40,41,42] have proposed decision-making algorithms utilising the SAMe-TT_2_R_2_ score to aid in the choice between warfarin and NOACs, and recommending reassessment of choice dependent on TTR. However anticoagulant choice for specific patients, e.g., those with valvular AF, may be limited to warfarin but the utility of the SAMe-TT_2_R_2_ model in these patients remains by identifying potentially poor warfarin control. Interestingly, the original SAMe-TT_2_R_2_ model simply suggested a score ≥ 2 identifies patients who may require additional intervention to achieve acceptable warfarin control [13]. Esteve-Pastor et al. [40] highlighted that interventions could include monitoring by anticoagulation clinics to impact the overall TTR predicted by the SAMe-TT_2_R_2_ model. Our study reinforces this as the mean TTR of patients with a SAMe-TT_2_R_2_ score of ≥ 2 was 15% higher when managed by the WCP as opposed to the GP. Thus, the mean TTR of patients with a SAMe-TT_2_R_2_ score ≥ 2 may benefit from the additional monitoring and/or interventions provided by dedicated WCPs.

This study was retrospective in nature and whilst this enabled unbiased correlation of TTR and SAMe-TT_2_R_2_ score, it limited the ability to analyse other potentially impacting factors on TTR, e.g., compliance. Another limitation was that events, such as bleeds and stroke, were not definitively reported so analyses correlating predicted TTR to outcomes could not be performed. In this study, data regarding race was not available and data on tobacco use minimal, which limited the ability to investigate the impact on TTR by these variables. As a result, the sample size of patients with a SAMe-TT_2_R_2_ score ≥ 2 was small, so further studies including more patients with higher SAMe-TT_2_R_2_ scores are necessary to determine whether the results of this study are generalisable to the entire Australian population.

## 5. Conclusions

In conclusion, this study applied the SAMe-TT_2_R_2_ model without race as a factor to an Australian population. A SAMe-TT_2_R_2_ score ≥ 2 was associated with significantly lower TTR in patients managed by the WCP and differentiated patients achieving a TTR below 65% in the subset of patients managed by their GP. This study suggests that, despite the absence of recorded race, the SAMe-TT_2_R_2_ model could assist Australian clinicians in identifying patients who may obtain reduced warfarin control.

## Figures and Tables

**Table 1:** Patient demographics in relation to variables in the predictive score for patients managed by the warfarin care program and by a general practitioner. Data is represented as number (percentage) and median (25th and 75th percentile) for HAS-BLED (Hypertension, Abnormal renal/liver function, Stroke history, Bleeding history, Labile International Normalised Ratio, Elderly, i.e., age > 65 years, Drugs/alcohol concurrently) and CHA_2_DS_2_VASc (Congestive heart failure, Hypertension, Age ≥ 75 years, Diabetes mellitus, Stroke/transient ischaemic attack, Vascular disease, Age 65-74 years, Sex – female) risk scores.

Patient Characteristic	Warfarin Care Program (n = 3911)	General Practitioner (n = 200)
**Gender**		
Female	1901 (48.6)	96 (48.0)
Male	2010 (51.4)	104 (52.0)
**Age**		
<60 years	150 (3.8)	2 (1.0)
≥60 years	3761 (96.2)	198 (99.0)
**Medical History**		
More than two of the following: hypertension, diabetes, coronary artery disease/myocardial infarction, peripheral arterial disease, congestive heart failure, previous stroke, pulmonary disease, hepatic or renal disease	254 (6.5)	37 (18.5)
**Concurrent Treatment Amiodarone**	220 (5.6)	10 (5.0)
**Tobacco Use (within 2 years)**	17 (0.4)	0 (0)
**Race**	N/A	N/A
**HAS-BLED**	1 (1–2)	2 (1–2)
**CHA_2_DS_2_VASc**	3 (2–4)	3 (2–4)

**Table 2:** Time in therapeutic range (TTR) in relation to SAMe-TT_2_R_2_ (Sex, Age, Medical history, Treatment, Tobacco use, Race - Caucasian) score for patients managed by a warfarin care program and by a general practitioner. Data is represented as number (percentage) for patients and mean (standard deviation) for time in therapeutic range with statistics shown for comparison to SAMe-TT_2_R_2_ score 0 or 0–1 when categorised.

	Warfarin Care Program (n = 3911)			General Practitioner (n = 200)		
**SAMe-TT_2_R_2_ Score**	**Patients**	**TTR**	***p*-value**	**Patients**	**TTR**	***p*-value**
**0**	1646 (42.1)	80.9 (9.4)		64 (32.0)	70.1 (15.4)	
**1**	1937 (49.5)	80.6 (9.5)	*p* = 0.344	109 (54.5)	68.8 (16.8)	*p* = 0.775
**2**	308 (7.9)	78.6 (10.4)	*p* < 0.001	27 (13.5)	63.6 (15.0)	*p* = 0.061
**3–4**	20 (0.5)	78.0 (14.0)	*p* = 0.367			
**Categorised Score**						
**0–1**	3583 (91.6)	80.9 (9.5)		173 (86.5)	69.3 (16.3)	
**≥2**	328 (8.4)	78.6 (10.7)	*p* < 0.001	27 (13.5)	63.6 (15.0)	*p* = 0.089
